# Pharmacology and mechanisms of apigenin in preventing osteoporosis

**DOI:** 10.3389/fphar.2024.1486646

**Published:** 2024-12-12

**Authors:** Sun Lin, Wang Yincang, Du Jiazhe, Xu Xilin, Xiaofeng Zhang

**Affiliations:** ^1^ Second Affiliated Hospital of Heilongjiang, University Of Chinese Medicine, Harbin, China; ^2^ The First Clinical Medical College, Zhejiang Chinese Medical University, Hangzhou, Zhejiang, China; ^3^ The Third Affiliated Hospital of Heilongjiang, University of Chinese Medicine, Harbin, China

**Keywords:** apigenin, osteoporosis, oxidative stress, osteoblasts, inflammatory factors

## Abstract

Osteoporosis (OP) stands as the most prevalent systemic skeletal condition associated with aging. The current clinical management of OP predominantly depends on anti-resorptive and anabolic agents. Nevertheless, prolonged use of some of these medications has been observed to reduce efficacy and elevate adverse effects. Given the necessity for sustained or even lifelong treatment of OP, the identification of drugs that are not only effective but also safe and cost-efficient is of utmost significance. As disease treatment paradigms continue to evolve and recent advancements in OP research come to light, certain plant-derived compounds have emerged, presenting notable benefits in the management of OP. This review primarily explores the pharmacological properties of apigenin and elucidates its therapeutic mechanisms in the context of OP. The insights provided herein aspire to offer a foundation for the judicious use of apigenin in forthcoming research, particularly within the scope of OP.

## 1 Introduction

Osteoporosis (OP) is a metabolic bone disorder characterized by a reduction in bone mass, compromised bone microarchitecture, and heightened bone fragility (Lane et al., 2000). OP is categorized into primary and secondary forms. Primary OP encompasses postmenopausal OP, age-related OP, and idiopathic OP (Glaser and Kaplan, 1997). Secondary OP is defined by identifiable etiological factors (Favero et al., 2023). The globally acknowledged standard for diagnosing OP is dual-energy X-ray absorptiometry, which considers a bone mineral density (BMD) score of 2.5 standard deviations or more below the mean for young, healthy populations as indicative of OP (Lorentzon, 2019). Clinical manifestations commonly include pain, fractures, and skeletal deformities (Lamichhane, 2005). A 2021 meta-analysis examining epidemiological trends in OP over the past 2 decades reported a global prevalence of approximately 18. 3%, with prevalence rates of 23.1% in women and 11.7% in men. The higher incidence in women is primarily attributed to postmenopausal estrogen reduction and endocrine metabolic disturbances (SALARI et al., 2021). Presently, the global population affected by OP exceeds 200 million. The increasing age of the population is expected to drive a rising trend in OP prevalence, with projections suggesting that by 2050, the number of individuals over 50 years old with OP will surpass 400 million globally (Ma et al., 2023). Additionally, the high bone fragility in OP patients increases the risk of fractures (Zhang et al., 2023). This condition leads to a diminished quality of life, elevated disability and mortality rates, and substantial economic and caregiving burdens on patients and healthcare systems (Tung et al., 2024; Srivastava and Deal, 2002).

Currently, the therapeutic strategies for OP are primarily divided into two major categories: a) Remodeling inhibitors, which encompass four types of bisphosphonates, selective estrogen receptor modulators, procalcitonin, estrogen, and denosumab; and b) Bone anabolic agents, which include abaloparatide, parathyroid hormone, and overlapping therapy (Porwal et al., 2021). Additionally, non-pharmacological interventions such as appropriate exercise, a balanced diet, and the cessation of smoking and alcohol consumption are recommended (Aibar-Almazán et al., 2022). Given the high costs and notable side effects associated with certain clinical medications, the pursuit of novel drugs remains imperative. Research has indicated that natural active compounds derived from fruits, vegetables, and medicinal plants hold significant promise in the prevention and treatment of OP through diverse mechanisms, potentially leading to the development of alternative therapies that are more cost-effective, have reduced side effects, and are suitable for long-term use (Li et al., 2023). Apigenin (API), a natural flavonoid compound (Hong et al., 2022), has been reported to exhibit a broad spectrum of pharmacological activities, including anti-inflammatory (Liang et al., 2023), neuroprotective and neurotrophic (Patil et al., 2014), and anti-oxidative stress (Seo et al., 2014) effects. This study aims to review the potential mechanisms and future prospects of API in the management of OP.

## 2 Biological characteristics of API

### 2.1 Origin of API

API, which is derived from the *Apium* genus within the Apiaceae family, is present in specific fruits, vegetables, and medicinal plants (Darabi et al., 2020). As a small flavonoid molecule, it predominantly exists in its glycosylated form and is classified as a secondary metabolite of plants (Madunic et al., 2018). The concentration of API in *Coriandrum sativum L*. surpasses that in *Apium graveolensL*. by 180-fold. Moreover, API functions as a critical active ingredient in various herbal remedies, including *Scutelaria baicalensis Georgi*, *Scutelaria barbata D*. *Don*, *Perilafrutescens Brit*, *Plantago asiatica*, and *Taraxacum oficinale* (Sara et al., 2020).

### 2.2 Physicochemical properties of API

API is presented as light yellow, needle-like crystals. The chemical name of API is 4', 5, 7-trihydroxyflavone, with a molecular formula of C15H19O5. Hydroxyl groups are located at the C-5 and C-7 positions on the A ring and at the C-4′ position on the B ring. The relative molecular mass of API is 2 7 0. 2 4 Da, and its melting point falls within the range of 3 4 5°C to 350°C. While API is insoluble in water, it exhibits solubility in dimethyl sulfoxide, hot ethanol, and dilute alkali solutions. According to the Biopharmaceutics Classification System, it is categorized as a Class II compound (Darabi et al., 2020; Jang et al., 2022; DeRango-Adem et al., 2021), Figure 1 illustrates the structure of API.

**FIGURE 1:**
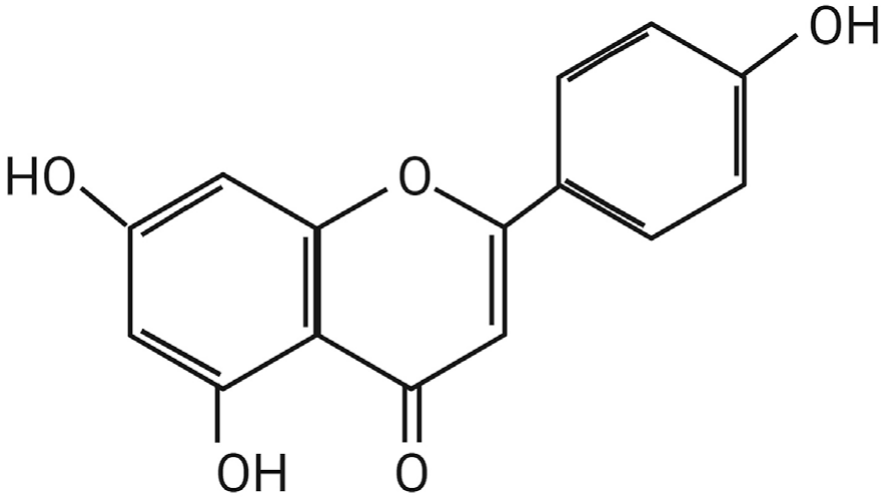
The chemical structure of Apigenin.

### 2.3 Biosynthesis of API

**FIGURE 2:**
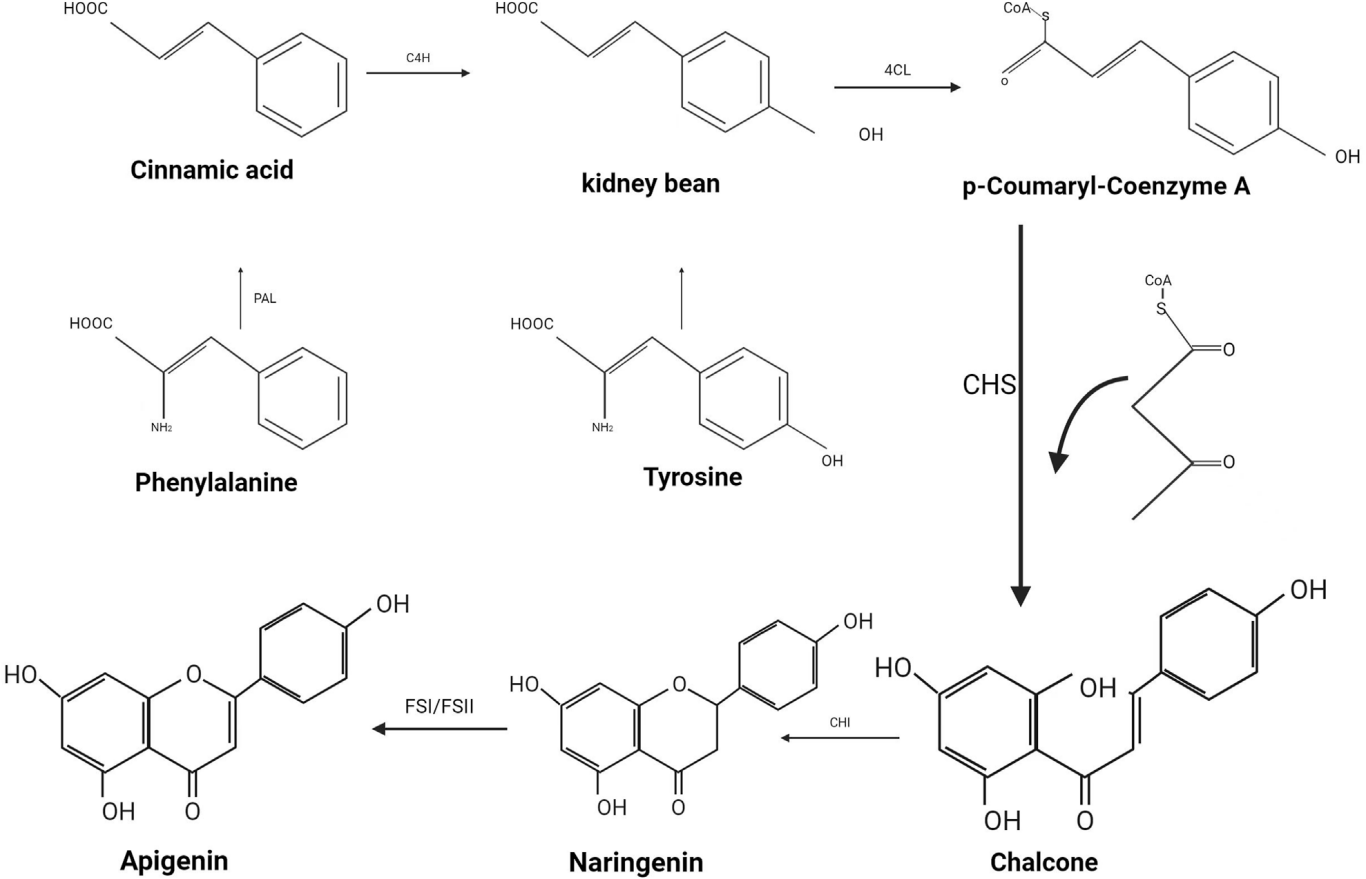
The production process of Apigenin. Biosynthesis process of API.

Flavonoids, a class of natural polyphenols, are characterized by a common structure that incorporates multiple potential molecular functionalities. The distinctive tricyclic alkaloid base is synthesized by the polyketide synthase family, enabling the formation of over 6, 000 distinct molecular structures through modifications such as hydroxylation, methoxylation, and glycosylation (Santos-buelga and Feliciano, 2017). From a biogenetic perspective, API is derived from the phenylpropanoid pathway, a product generated during the synthesis of phenylalanine and tyrosine (as illustrated in Figure 2). Phenylalanine, under non-oxidative deamination by phenylalanine ammonia-lyase, is converted into cinnamic acid, which subsequently undergoes oxidation at the C4 position by cinnamic acid-4-hydroxylase to yield coumaric acid. Similarly, tyrosine, through deamination, produces coumaric acid. The enzyme 4-coumaric acid-coenzyme A ligase reacts with coumaric acid to produce p-coumaroyl-CoA, which then combines with malonyl-CoA residues and is catalyzed by chalcone synthase to form chalcone. This chalcone is subsequently isomerized by chalcone isomerase to yield naringenin. Finally, flavonoid synthase (FSI/FSII) facilitates the oxidation of naringenin, culminating in the formation of API (Salehi et al., 2019).

## 3 Molecular mechanisms of API in OP

### 3.1 Effects of API on osteogenic differentiation of human bone marrow mesenchymal stem cells (hBMSCs)

hBMSCs serve as crucial sources for osteocyte precursors and play a significant role in bone remodeling, with the ability to induce osteogenic differentiation. Nevertheless, this process is often hindered or disrupted by various factors or receptors, which can inhibit or terminate the osteogenic differentiation of hBMSCs. As a result, a reduction in hBMSCs can lead to the development of OP and is a critical factor contributing to the failure of OP treatment (Li et al., 2023; F et al., 2020).

The Runt-related transcription factor 2 (RUNX2) gene is pivotal in regulating the differentiation of hBMSCs into osteoblasts (OB) and their subsequent maturation during skeletal development. API has been demonstrated to activate c-Jun N-terminal kinase and p38 mitogen-activated protein kinase, leading to the upregulation of osteopontin messenger RNA (mRNA) and the transcription factor Runx2, thereby promoting osteogenesis in hBMSCs. The expression of the Runx2 gene signifies the commencement of OB differentiation. Asadi et al. (2022) identified that API can counteract the inhibitory effects of lipopolysaccharide and palmitic acid (PA) on osteogenesis. It achieves this by diminishing the expression of NOD-like receptor protein three and reducing the activity of cysteinyl aspartate-specific proteinase-1 (caspase-1), while concurrently enhancing the osteogenic capacity of hBMSCs.

### 3.2 Wnt/β-catenin signaling pathway

Recent research has demonstrated that the Wnt/β-catenin signaling pathway plays a regulatory role in the growth and metabolism of articular cartilage, OB, and synovial cells. This pathway functions as a pivotal regulator of OB formation, differentiation, and OC activity, and is intimately linked with bone proliferation (U et al., 2016; Y et al., 2016). The Wnt/β-catenin pathway modulates bone cell activity by elevating both the level and activity of intracellular β-catenin (García-García. et al., 2022). Typically, phosphorylated β-catenin undergoes degradation, yet Wnt proteins can activate Frizzled receptors, which in turn decrease β-catenin phosphorylation, thereby enhancing its stability within the cytoplasm. The stabilized β-catenin then translocates to the nucleus, where, influenced by transcription factors such as TCF/LEF, it induces the expression of Wnt target genes (Tang et al., 2021).

**FIGURE 3:**
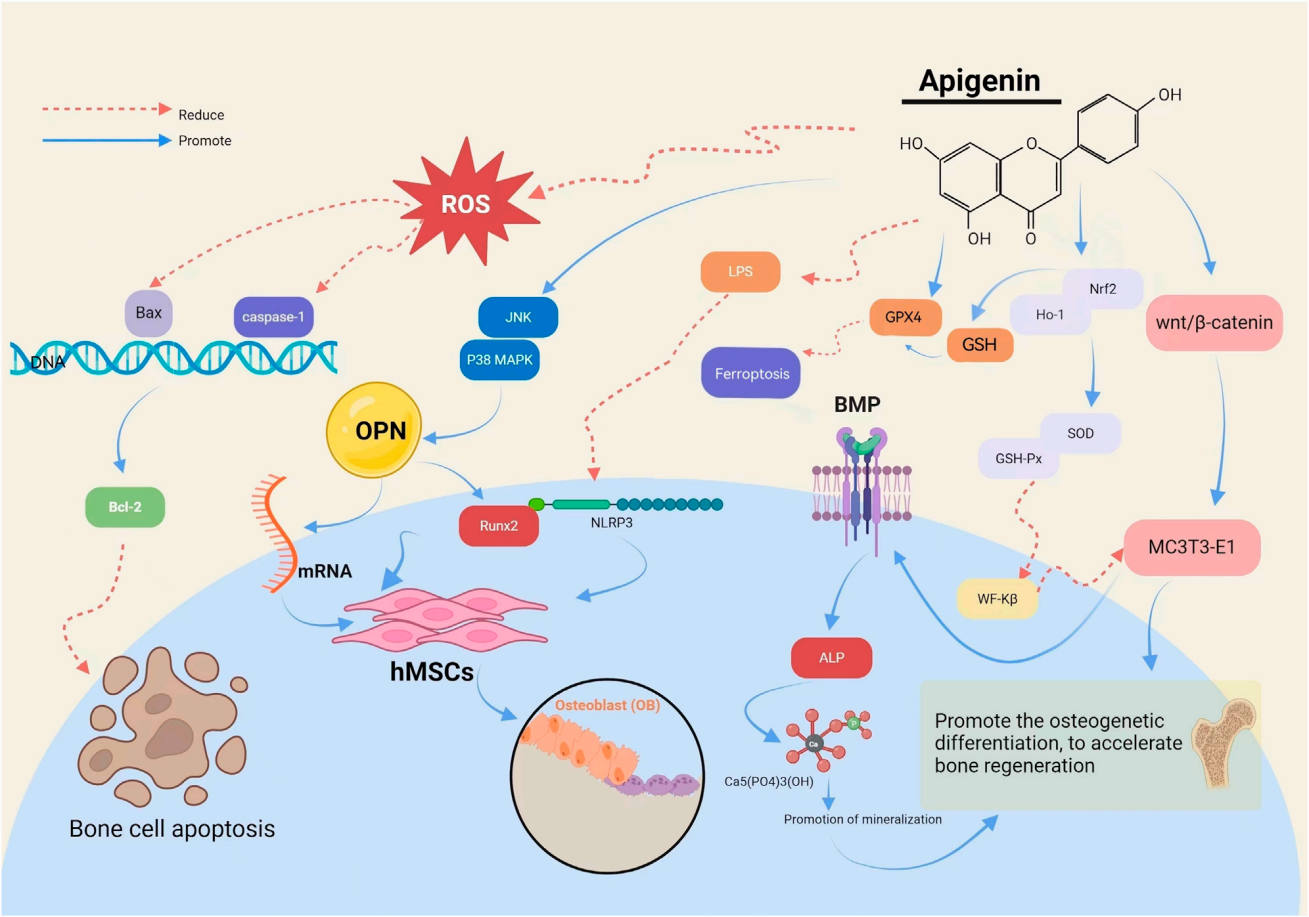
Biosynthesis process of API.

API (Pan et al., 2021) has been shown to elevate β-catenin protein levels and activate the Wnt/β-catenin pathway, thereby promoting osteogenic differentiation in MC3T3-E1 cells. This activation results in the accumulation of β-catenin within the cytoplasm and aids in the translocation of transcription activator family members to the nucleus, initiating osteogenic differentiation and bone regeneration. Furthermore, it has been observed (Jiao et al., 2019) that API can inhibit Wnt/β-catenin signaling in a concentration-dependent manner, leading to the downregulation of Wnt target genes such as the myelocytomatosis viral oncogene homolog, recombinant axis inhibition protein 2 (AXIN2), and recombinant cyclin D1 (CCND1), thereby reducing tumor cell invasion as illustrated in Figure 3.

### 3.3 Mediating the OS pathway

OS is intricately linked to the dysregulation of OB and OC functions, which ultimately contributes to OP. OS can impair the function of hBMSCs and OBs through various pathways, thereby diminishing bone differentiation. A sustained imbalance between antioxidant defenses and reactive oxygen species (ROS) may result in elevated lipid peroxidation, a reduction in antioxidant enzymes, and accelerated OB apoptosis (Kimball JS. et al., 2021; Geng et al., 2019; Da Silva et al., 2010), The nuclear factor E2-related factor 2 (Nrf2) plays a pivotal role in regulating the OS response. When Nrf2 binds to the antioxidant response element, it triggers the expression of endogenous antioxidant enzymes, such as superoxide dismutase (SOD) and glutathione peroxidase (GSH-Px), thereby enhancing the body’s antioxidant capacity (An and Shang, 2018). Heme Oxygenase-1 (HO-1), a downstream target gene of Nrf2, catalyzes the degradation of heme, producing biliverdin, which bolsters cellular defense mechanisms and mitigates OS (Chen et al., 2019). Elevated intracellular ROS levels can activate FoxO family proteins, which interact with β-catenin, leading to its increased nuclear translocation. In the nucleus, this complex binds to transcription factors, exerting transcriptional regulation. Within the Wnt pathway, this process upregulates the expression of antioxidant enzymes like SOD and catalase (CAT), thereby reducing OS, inhibiting the osteogenic differentiation of hBMSCs, and decreasing OB-mediated bone formation (Kimball J. S. et al., 2021).

API has been demonstrated to upregulate the expression of Nrf2 and HO-1, increase the activities of SOD and GSH-Px (Dang et al., 2020; Pedersini et al., 2020), and inhibit the nuclear translocation of nuclear factor Kappa-B (NF-κB) (Li et al., 2020). These actions collectively contribute to the reduction of oxidative cellular damage in MC3T3-E1 cells and the improvement of bone repair and OS-related bone metabolism (Jung, 2014). By lowering ROS levels, API plays a regulatory role in OS and inhibits lipid peroxidation. Under H2O2 treatment, API has been observed to modulate the protein levels of Nrf2, reduce ROS levels, and enhance CAT activity (Chen et al., 2019b), thereby alleviating OS-induced damage.

### 3.4 Promotion of mineralization

The mineralization process necessitates the involvement of proteins such as recombinant Annexin (Anx) and tissue nonspecific alkaline phosphatase (TNAP) (Bozycki et al., 2021). TNAP, which can exist in a soluble form, is responsible for hydrolyzing inorganic pyrophosphate (PPi) into phosphate (Pi). In conjunction with recombinant ectonucleotide pyrophosphatase/phosphodiesterase 1, TNAP hydrolyzes adenosine triphosphate to produce PPi, serving as the primary regulator of PPi/Pi balance. The conversion of PPi to Pi by TNAP is essential for the mineralization process (Mroczek et al., 2022). Anx, a protein that binds to calcium ions and phospholipids, is partially localized within the matrix vesicle (MV) lumen or associated with the inner or outer surface of the MV membrane, which is abundant in phosphatidylserine. Recombinant Annexin A6 (AnxA6), comprising eight domains, forms transmembrane ion channels under acidic pH conditions, allowing calcium ions to pass through the membrane and act as activators or inhibitors of mineral formation (Veschi et al., 2020; Takegahara et al., 2024).

API facilitates the differentiation of cells into mature, bone-forming OB (Azam et al., 2023). It upregulates OB differentiation genes, including bone morphogenetic protein (BMP), in mouse MC3T3-E1 OBs. BMP, a multifunctional growth factor, plays a crucial role in regulating alkaline phosphatase (ALP) activity. ALP, an enzyme secreted by OBs, serves as an early marker of osteoblastic differentiation and functional status by reflecting the degree of OB differentiation through its expression level in cells. This enzyme hydrolyzes pyrophosphate into phosphate, a process vital for hydroxyapatite formation. The increase in ALP activity raises the local phosphate concentration, which leads to mineralization through hydroxyapatite crystallization (Bottini et al., 2018). The formation of mineralized nodules marks the late stage of OB differentiation, representing the phase where OBs are fully mature and actively engaged in bone formation, and serves as a morphological manifestation of osteogenic function. In the API-treated group, a significant increase in the number of calcium nodules was observed, with these nodules appearing darker in color and showing a concentration-dependent enhancement. This observation indicates that API has a pronounced effect on promoting cell mineralization. Furthermore, studies have suggested that API can regulate the mineralization process in human bone cells *in vitro* by modulating AnxA6 and TNAP in MVs (Mroczek et al., 2022).

### 3.5 Mechanisms of API-mediated inhibition of OC-induced bone resorption

OC, which are terminally differentiated cells, predominantly contribute to bone resorption. The differentiation of OC can be induced through receptor activators for nuclear factor-κB ligand (RANKL) and macrophage colony-stimulating factor (M-CSF) (Reinholz et al., 2000). OC activity is modulated by the nuclear factor of activated T cells 1 and the oncogene Fos, enabling bone resorption by secreting enzymes such as tartrate-resistant acid phosphatase (TRAP), cathepsin K (CTSK), and matrix metalloproteinase 9, which degrade the bone matrix (Yanan et al., 2023). Excessive OC activation results in accelerated bone loss and reduced bone density, which are critical factors in the development of OP. Thus, the reduction of OC over-differentiation is crucial for controlling bone loss.

#### 3.5.1 Regulatory inflammatory factors

The generation of OB and the dynamic modulation of anti-OB cytokines are pivotal for the preservation of skeletal homeostasis. Within the phase of macrophage polarization induction, inflammatory mediators facilitate OC differentiation via pro-inflammatory mechanisms, hastening bone resorption and precipitating OP. Tumor necrosis factor α (TNF-α) instigates the translocation of NF-κB into the nucleus, thereby perturbing the equilibrium of the RANK-RANKL axis, augmenting OC activity, and upregulating pro-inflammatory genes linked to RANK, consequently advancing OC differentiation (Wang et al., 2018). Moreover, interleukin-1β and interleukin-6 contribute to the differentiation and maturation of OCs through RANKL, subsequently inducing bone resorption (Mo et al., 2024). RANKL, a pivotal cytokine for the differentiation and activation of OCs, facilitates the activation of the NF-κB receptor activator (RANK)/RANKL signaling pathway, thereby enhancing OC differentiation, resulting in an excessive increase in OCs and intensifying bone degradation (Ren et al., 2022).

The NF-κB signaling pathway and its consequent impact on bone tissue are markedly inhibited by API. Studies focused on the anti-psoriasis effects of API have demonstrated its ability to diminish the expression and secretion of pro-inflammatory cytokines, including recombinant chemokine C-C-Motif receptor 6 (CCR6), interleukin-17 (IL-17), and NF-κB, through modulation of the interleukin-23/IL-17/interleukin-22 (IL-22) axis. This, in turn, results in the inhibition of OC activation and a subsequent reduction in bone degradation (Rahmani et al., 2022).

#### 3.5.2 RANKL/RANK/osteoprotegerin (OPG) signaling pathway

The RANKL/RANK/OPG signaling pathway plays a pivotal role in OC formation and skeletal remodeling. In the presence of M-CSF, RANK engages with the C-terminus of RANKL, thereby facilitating the transcription and expression of downstream OC-specific genes through tumor necrosis factor receptor-associated factors. This interaction initiates signaling cascades that involve NF-κB, adenosine 3′,5′-cyclic monophosphate, and recombinant nuclear factor of activated T-cells cytoplasmic 1 (NFATc1), which, in turn, induce the expression of proteins such as TRAP, integrin beta 3, and CTSK, governing OC differentiation and bone resorption (Zhang et al., 2019). OPG, which is secreted by OB, functions as a decoy receptor for RANKL, thereby inhibiting the interaction between RANKL and RANK. Such inhibition reduces the formation and maturation of OCs, ultimately leading to decreased OC activity (Udagawa et al., 2021).

API has been reported to lower serum levels of bone Gla-protein and ALP while also diminishing the mRNA and protein expression levels of OPG in femoral tissue, thereby expediting bone healing (Zhang et al., 2023). Moreover, API has the capacity to activate the PI3K/Akt pathway and upregulate the expression of RUNX2 and OPG while concurrently inhibiting RANKL expression. This results in an indirect increase in the OPG/RANKL ratio, restoring the equilibrium of the RANKL/OPG system and thereby augmenting the healing potential of bones damaged under OP conditions (Feng et al., 2023).

## 4 Other effects of API on bone metabolism

### 4.1 Estrogen receptor signaling pathway

A reduction in **Estrogen** levels disrupts the equilibrium between bone resorption and formation, resulting in diminished bone strength, an elevated risk of fragility fractures, and decreased BMD. This reduction in **Estrogen** levels fosters OC differentiation and activity, which may initiate the onset of OP (Słupski et al., 2021). **Estrogen** is also widely employed in the clinical management of skeletal disorders due to its role in regulating bone homeostasis through both bone formation and resorption. Phytoestrogens, which share structural and functional similarities with **Estrogen**, particularly in receptor binding, can emulate the effects of **Estrogen** while mitigating potential side effects. Furthermore, phytoestrogens have the capacity to enhance calcium absorption via **Estrogen** receptor pathways in intestinal cells, thereby affecting bone remodeling (Xu et al., 2016).

API, a phytoestrogen with a chemical structure akin to 17β-estradiol (E2), commonly prescribed for postmenopausal OP treatment, demonstrates bidirectional activity by functioning both as an **Estrogen**-mimetic and an **Estrogen** inhibitor, thereby potentially providing protection against OP (Long et al., 2008; Yang et al., 2015; Lu et al., 2021). Moreover, API has been demonstrated to interact with OP-related signaling pathways, such as the mitogen-activated protein kinase and Wnt pathways. It enhances E receptor expression in OC and OB, increases ALP activity in OB, augments bone collagen content, and decreases the secretion of OC-promoting factors induced by interferon-γ and TNF-α. These effects collectively contribute to bone formation, regulation of bone resorption, and improvement in bone turnover (Tantowi et al., 2020).

### 4.2 Improvement of gut microbiota

With the advancement of research into gut microbiota, orthopedic researchers have increasingly examined the correlation between gut microbiota and orthopedic diseases (Awuti et al., 2020). It has been observed that gut microbiota can upregulate the expression of OPG and mammalian targets of rapamycin transcription factors, thereby modulating osteoclast differentiation (Schepper et al., 2020). The reduction in BMD is strongly linked to alterations in the abundance of specific bacterial populations, with their mediated catabolic effects being closely associated with pathological bone diseases (Pedersini et al., 2020; Li et al., 2021). Dysbiosis of the gut microbiota can disrupt the pH balance within the intestine, influencing the absorption of calcium, phosphorus, and vitamin D. It can also elevate intestinal permeability and compromise the barrier function of the intestinal epithelium, leading to bone loss and trabecular bone fractures (Guan et al., 2020).

API has been demonstrated to remodel the gut microbiota, mitigate inflammation, fortify intestinal immune barriers, and sustain intestinal homeostasis (Fu et al., 2022). It modulates bacterial cell membrane permeability and impacts bacterial cell wall integrity, thereby curbing the proliferation of *Staphylococcus aureus*, *Escherichia coli*, and *Candida albicans*, leading to an improvement in the intestinal flora (Sato et al., 2000). This process supports the maintenance of overall health. A balanced gut microbiome facilitates nutrient absorption, strengthens the body’s immune system and disease resistance, while also aiding recovery from skeletal diseases as illustrated in Table 1.

**TABLE 1:** To study the therapeutic model and mechanism of apigenin on osteoporosis in vitro and in vivo.

Type of experiment	Animal/cell	Mechanism	action	References
In vivo experiments	Rats	Reshaping the intestinal bacterial community, alleviating inflammation and maintaining intestinal homeostasis	It can improve the body's immunity and is beneficial to the rehabilitation of bone diseases	Fu et al., 2022
	Rats	Activation of PI3K/Akt pathway can up-regulate the expression of Runx2 and OPG, and increase the OPG/RANKL ratio	Repair the healing ability of damaged bone in the OP state	Feng et al., 2023
	Mice	Decreased STAT3 differentiation to express CD36 decreased PPAR-γ expression, and activated AMPK	Reduce fat cells and inhibit lipogenesis, which is beneficial to bone health	Su et al., 2020
	Mice	Activation of GPX4 can activate Nrf2 expression and promote the increase of GSH level	Inhibition of ferroptosis	Zhang et al., 2020; Zhao et al., 2020; Chen et al., 2024
In vitro experiments	hMSCs	JNK and p38 MAPK were activated, and the expression of mRNA and Runx2 was up-regulated	It promotes osteogenesis	Asadi et al., 2022
	MC3T3-E1	Increases β-catenin protein levels, via the Wnt/β-catenin pathway	It stimulates osteogenic differentiation and bone regeneration	Pan et al., 2021
	MC3T3-E1	The expression of Nrf2 and HO-1 was up-regulated, and the activities of SOD and GSH-Px were increased	Enhanced bone repair and bone metabolism associated with oxidative stress	Dang et al., 2020; Pedersini et al., 2020; Li et al., 2020; Jung, 2014; Chen et al., 2019
	MC3T3-E1	Upregulating BMP, regulating ALP activity, and decomposing pyrophosphate to phosphate	Promotion of mineralization	Azam et al., 2023; Bottini et al., 2018

### 4.3 Reduction of adipocyte count

Adipocytes and OB both originate from mesenchymal cell precursors derived from bone marrow. Adipocytes exhibit the capability to self-enhance their differentiation, potentially influencing the quantity of OB in the bone marrow and subsequently impacting BMD (Proietto, , 2020; Greco et al., 2010).

API is capable of binding to the non-phosphorylated form of the signal transducer and activator of transcription 3 (STAT3), which results in a decrease in the expression of the STAT3 differentiation target gene cluster of differentiation 36 (CD36) and also reduces the expression of peroxisome proliferator-activated receptor γ (PPAR-γ). Consequently, the lowered expression of CD36 and PPAR-γ serves to inhibit adipogenesis (Su et al., 2020). Moreover, API can activate adenosine monophosphate-activated protein kinase, which leads to a downregulation of genes associated with lipogenesis and lipolysis. This process diminishes PA-induced lipid accumulation in HepG2 cells, thereby reducing lipid content and suppressing adipocyte differentiation (Lu et al., 2019). The potential of API in decreasing adipocytes and inhibiting adipogenesis is evident, indirectly supporting skeletal health and possibly exerting an influence on OP. However, current research is limited, necessitating further exploration and validation of these specific mechanisms.

### 4.4 Ferroptosis

Recent investigations have demonstrated that ferroptosis constitutes an iron-dependent form of cell death characterized by iron accumulation and lipid peroxidation. This phenomenon influences the proliferation and differentiation of osteoblasts (OBs), resulting in bone loss and being recognized as one of the common risk factors for osteoporosis (OP) (Luo et al., 2022; Jiang et al., 2022). Nuclear factor erythroid 2-related factor 2 (Nrf2) serves as a crucial regulator of lipid peroxidation and ferroptosis. Glutathione peroxidase 4 (GPX4) is a specific marker of ferroptosis. When the activity of GPX4 is inhibited, the antioxidant capacity of cells is diminished. Excessive Fe3+ generates a substantial amount of reactive oxygen species (ROS) through the Fenton reaction, leading to the accumulation of lipid peroxides, causing oxidative damage and subsequently inducing the occurrence of ferroptosis (Zhang et al., 2024; Che et al., 2020). The nuclear transcription of Nrf2 regulates the expression of GPX4 and the antioxidant capacity of cells, activates cell membrane iron transporter proteins, and balances intracellular iron concentration (Bao et al., 2023; Ma et al., 2020; Deng et al., 2024).

Di(2-ethylhexyl) phthalate (DEHP) is an artificial persistent organic pollutant (POP) capable of inducing ferroptosis-like injury. API can activate GPX4, inhibit intracellular iron accumulation, and exert a protective role against DEHP-induced ferroptosis (Han et al., 2022). The potential role of apigenin in regulating ferroptosis has been established in other pathological studies. For instance, API can activate the expression of Nrf2, promote the elevation of intracellular glutathione (GSH) level and scavenge it after binding to ROS, inhibiting the occurrence of lipid peroxidation during ferroptosis. Meanwhile, a high concentration of GSH helps maintain the activity of GPX4, which is capable of reducing lipid peroxides to harmless lipols, further preventing the development of ferroptosis (Zhang et al., 2020; Zhao et al., 2020; Chen et al., 2024). It has also been shown that API can downregulate the oxidative stress gene ATF3 and upregulate GPX4 in non-obese diabetic (NOD) mice, inhibiting ferroptosis in salivary gland epithelial cells (SGECs) (Liu et al., 2024). This suggests that in OP, API may slow down the degradation of bone matrix and the decrease in bone mineral density (BMD) by inhibiting ferroptosis.

## 5 Advantages of apigenin in the treatment of osteoporosis

### 5.1 Natural sources with fewer side effects

API is a naturally occurring flavonoid found in fruits and vegetables, exhibiting low toxicity and minimal side effects. In comparison to common therapeutic agents such as bisphosphonates (BPS) (Ng et al., 2004), it can inhibit osteoclast activity and is well tolerated. However, BPS can induce osteonecrosis of the jaw, aseptic inflammatory responses, and increase leukocyte-endothelial cell interactions, leading to the development of secondary infections and impaired healing (Ting et al., 2007). Denosumab has been demonstrated to increase BMD and reduce fracture risk in patients with OP, but there is a rapid rebound of the bone resorption effects after discontinuing the drug (Zhou et al., 2022; Alfaleh et al., 2022). Tetracyclines (TCs) are clinical broad-spectrum antibiotics that can be deposited in bone tissue and bind to calcium ions in the main components of bone. Due to their excellent osteotropic properties, they are utilized as a targeting moiety of bone tissue for drug delivery to the target site to achieve targeted therapy (Seydi et al., 2016a). It has been found (Hicks et al., 2017) that TCs cause tooth staining and lead to enamel hypoplasia during calcification. Additionally, calcium and vitamin D supplementation are routinely paired for the treatment of OP, but calcium supplementation alone cannot compensate for renal calcium loss, and the increased circulating calcium load may lead to additional bone deposition (de Campos et al., 2023). Hormone replacement therapy is an effective treatment for postmenopausal OP. Although it is effective in improving bone density, long-term use of estrogenic medications induces the development of hypertension and edema, as well as increasing the risk of breast cancer, endometrial cancer, and blood clots (Vigneswaran and Hamoda, 2022). In contrast, apigenin, as a plant extract with estrogen-like effects, can reduce drug dependence and hormone-related risks, presenting itself as a safer alternative choice.

### 5.2 Multi-target mechanisms of action

As a natural flavonoid, API exhibits a multilevel and multi-target mechanism of action in osteoporosis treatment, with significant potential for preclinical application.API significantly promotes the differentiation of hBMSCs to osteoblasts and enhances their osteogenic capacity through the activation of key osteogenic signalling pathways (e.g., JNK and p3 8 MAPK) and the upregulation of expression of osteogenic genes Runx2 and OPN . In addition, API also plays an important role in inhibiting osteoclast-mediated bone resorption by interfering with the activities of NF- κ B and RANKL/RANK/OPG signalling axes, reducing osteoclast generation and maturation, thus effectively preventing bone loss.

Oxidative stress is considered an important pathological mechanism of osteoporosis. API reduces the damage of oxidative stress on osteoblasts, maintains osteoblast activity, and prevents excessive degradation of bone matrix by activating the Nrf2/HO-1 antioxidant signalling pathway and increasing the activity of SOD and GSH-Px. API, as a potent antioxidant and anti-inflammatory agent, can effectively mitigate the damage of oxidative stress and inflammation on bone tissue. As a potent antioxidant and anti-inflammatory agent, API is effective in mitigating oxidative stress and inflammation damage to bone tissue, and has a more comprehensive therapeutic potential to alleviate underlying pathological factors (e.g . , inflammation) while mitigating the underlying pathological factors (e.g., inflammation) than some conventional drugs that only target bone resorption or bone formation.API is also able to enhance the efficiency of mineralisation by increasing the inward flow of calcium ions in mineralisation through the upregulation of ALP activity, which promotes the calcific deposition of bone matrix. In addition, API also maintains the balance of bone metabolism by regulating the stability of the intestinal flora, reduces the number and differentiation of adipocytes, and prevents bone marrow fat from negatively affecting the osteogenesis process, further supporting the maintenance and enhancement of bone density. This mechanism of indirectly affecting bone health through the microbiota is not possible with some of the conventional treatments currently available, and has the potential to provide a new pathway for the treatment of osteoporosis.

Overall, the synergistic effects of API on multi-target signalling pathways enable it to exhibit unique advantages in maintaining bone metabolic homeostasis, resisting oxidative stress and regulating the balance of bone production and resorption, and these properties provide potential clinical applications and directions for further research on APIs in the treatment of osteoporosis.

### 5.3 Potential to improve bioavailability

The bioavailability of API is influenced by factors such as food matrix, bioaccessibility, digestibility, molecular structure, and metabolic enzymes (Waheed et al., 2023). With a solubility of 0.001–1 .63 mg/mL in non-polar solvents and 2. 16 μg/mL in water, oral bioavailability is low, limiting its clinical application (Zhang et al., 2012) ^.^ The conversion of apigenin to macromolecules such as glucosides in the intestinal mucosa is an important factor affecting its biodistribution and reducing its net intestinal absorption (Wang et al., 2016). Methylation, sulfonation, and glucuronidation of apigenin also affect its bioavailability. The Caco-2 cell monolayer system revealed that API can be a substrate for glucuronidation of uridine 5′-diphosphate o-glucuronosyltransferase present in the intestinal epithelium. The permeability coefficient of API is at 10–5 cm/s, it has high lipophilicity, and it is well absorbed in the intestinal mucosa and eliminated slowly in serum (Awad et al., 2023; Ng et al., 2004). After discontinuation of the drug, API levels peaked after 3.9 h, with recovery rates of 16 .6% in urine and 28 .6% in feces (Ting et al., 2007). Some researchers have found liposomes, self-microemulsifying drug delivery systems, nanocrystalline gel formulations, and many other strategies and techniques to enhance its bioavailability. API-loaded polymer micelles can increase its solubility and oral bioavailability by 148 and 4 .03 folds, respectively, compared to API alone, suggesting that polymer films can act as a delivery system for API (Zhou et al., 2022). This provides greater advantages for apigenin in clinical applications and may confer greater efficacy without increasing toxicity and side effects.

### 5.4 Synergistic effects in combination with other natural medicines

API can exhibit synergistic effects with other natural drugs or nutrients. Raloxifene is a phenothiophene compound with estrogenic effects in regulating bone levels, which can increase bone mineral density and reduce bone loss. API combined with raloxifene can reduce the first-pass metabolism of raloxifene and its glucuronidation and sulfation in the intestinal tract, reduce the intestinal metabolism and outward transfer function, and improve drug utilization and enhance the efficacy of treatment for postmenopausal OP (Chen et al., 2010). The combination of API with paclitaxel inhibits superoxide dismutase (SOD) activity, promotes ROS-mediated cleavage of caspase-2, and attenuates to a certain extent the toxicity of paclitaxel (Young-Ah et al., 2015) This multi-component combination therapy may enhance therapeutic efficacy while reducing the problems of dependence and resistance to a single agent, a potential advantage not available with existing single-agent therapies.

Apigenin derivatives also have therapeutic effects on osteoporosis. Studies have shown that Isovitexin, a natural derivative of apigenin, effectively promotes bone production through activation of mitochondrial biosynthesis and respiration in osteoblasts in an estrogen-deficient mouse model, demonstrating its potential for osteoporosis prevention and treatment (Isika et al., 2020). Isovitexin has been shown to interact with adiponectin receptors (AdipoRs), thereby triggering the upregulation of peroxisome proliferator-activated receptor γ coactivator 1 α (PGC - 1 α), a key mitochondrial biogenesis factor within osteoblasts. Concomitantly, this interaction promotes oxidative phosphorylation (OxPhos) and ATP synthesis, ultimately leading to enhanced osteoblast differentiation (Pal et al., 2021) In studies utilizing the femur osteotomy model in adult mice and the ovariectomized osteopenic mouse model, it was observed that Isovitexin at doses of 2.5 and 5 mg/kg markedly augmented bone formation at the osteotomy site (Pal et al., 2022). Another study found that both API and rutin derivatives promoted osteogenic differentiation of human bone marrow stromal stem cells (hBMSCs). These compounds effectively enhanced osteogenic activity by activating signaling pathways such as JNK and p38 MAPK. Especially in age-related osteoporosis, these derivatives showed high therapeutic potential in reducing cellular aging and bone loss (Ali et al., 2024). In addition, it has been reported (Isika et al., 2020) that a series of apigenin amide derivatives have been validated for their potential to enhance osteogenic activity by molecular docking simulations.

These compounds are designed to enhance the stability and activity of apigenin in bone tissue, thus improving its effect in anti-bone resorption and promotion of bone formation. These studies provide a new perspective for the application of apigenin derivatives in the treatment of osteoporosis.

### 5.5 Multiple prevention and treatment effects on other chronic diseases

In addition to bone protection, API have shown potential in combating some chronic diseases such as cardiovascular disease. There is a correlation between osteoporosis and cardiovascular disease, and many people with osteoporosis also suffer from cardiovascular problems. API have been widely studied for their cardiovascular protective effects, improving vascular health, anti-inflammatory, and antioxidant properties. Compared to existing treatments, APIs may provide additional health benefits to the cardiovascular system while protecting bone. In recent years, API have even gained attention as a common ingredient in health-promoting, balanced diets. With its lower intrinsic toxicity to normal and cancerous cells compared to other structurally related flavonoids, apigenin has been suggested as a complementary therapeutic agent for patients with hepatocellular carcinoma (Seydi et al., 2016b).

## 6 Conclusion

The increasing incidence of OP each year imposes a considerable burden on various patient populations and their families, underscoring the need for more effective intervention strategies. The pathogenesis of OP is intricately linked with hormonal imbalances, cellular autophagy, ferroptosis, OS, and gut microbiota dysbiosis. These pathological factors may operate independently or in synergy, influencing the growth, differentiation, and apoptosis of OB, OC, and osteocytes through the modulation of distinct signaling pathways. However, the precise mechanisms remain only partially understood. As research advances, additional pathogenic factors associated with OP continue to be identified. In terms of treatment, a substantial body of research has shown that active components of Chinese herbal medicines and traditional Chinese medicine formulations can effectively manage OP by modulating hormone levels, influencing cellular autophagy, inhibiting ferroptosis, counteracting OS responses, and maintaining gut microbiota balance. API displays a range of anti-osteoporotic effects, including the promotion of bone formation, reduction of bone resorption, inhibition of inflammation, antioxidant properties, and estrogen-like activities. These attributes render API a promising candidate for maintaining skeletal health and combating OP. However, the therapeutic efficacy, optimal dosage, and safety profile of API in human subjects still require extensive clinical trials for further elucidation. The broad spectrum of bioactivities exhibited by API provides new perspectives and directions for the clinical management and prevention of OP. API holds significant potential in the prevention and treatment of OP, with considerable clinical application value and research significance.
